# Microstructure Controlling, Properties, and Thermodynamic Analysis of SiC Joints Brazed with Ni-Ti Fillers

**DOI:** 10.3390/ma18122816

**Published:** 2025-06-16

**Authors:** Ming Li, Zihao Liu, Jiazhen Yan, Haojiang Shi, Jiang Wu, Renxin Li, Huabei Peng, Ruiqian Zhang, Jiacheng Shang

**Affiliations:** 1 Science and Technology on Reactor Fuel and Materials Laboratory, Nuclear Power Institute of China, Chengdu 610213, Chinamyzxshj@126.com (H.S.); zhang_ruiqian@126.com (R.Z.);; 2 School of Mechanical Engineering, Sichuan University, No. 24 South Rd, Yihuan Rd, Wuhou District, Chengdu 610065, China; lzihao2702@163.com (Z.L.); wjiang@stu.scu.edu.cn (J.W.); penghuabei@scu.edu.cn (H.P.)

**Keywords:** SiC ceramics, Ni-Ti brazing filler, microstructure controlling, joint properties, thermodynamic analysis

## Abstract

Silicon carbide (SiC) ceramics were brazed with Ni-Ti fillers at 1350 °C for 10 min. The experimental results show that with the increase in Ti content in the fillers, the interface layer composed of Ni_2_Si, Ni_3_Si_2_, graphite, and TiC becomes thinner due to the inhibition of the Ti/SiC reaction on the Ni/SiC reaction. When Ni-45Ti filler is used, TiC becomes the only phase of the interface layer in the brazing seam. The elimination of graphite improves the mechanical property of the joints. The shear strength of the SiC joints brazed by Ni-15Ti, Ni-30Ti, and Ni-45Ti fillers is 33 MPa, 92 MPa, and 125 MPa, respectively. From the point of thermodynamics, the calculated component point of the Ni/SiC reaction transition to the Ti/SiC reaction is *x*_Ti_ = 31 at.%. When the Ti content is higher than 31 at.%, the Δ*G*_Ni/SiC_ > Δ*G*_Ti/SiC_, and TiC will be preferentially generated at the interface. Therefore, the Ni/SiC reaction is inhibited and the harmful graphite is eliminated.

## 1. Introduction

With excellent mechanical and chemical properties at high temperatures, silicon carbide (SiC) has great potential applications in the fields of nuclear technology [1,2,3,4]. Especially after the Fukushima nuclear accident, SiC is regarded as an important candidate for an accident-tolerant fuel (ATF) cladding material. However, SiC_f_/SiC composite cladding is hard to fabricate, and is limited by the high brittleness and difficult processability [5,6]. Thus, the joint technology for SiC_f_/SiC composite cladding faces many difficulties, and the progress of research on SiC joined with SiC or other materials is relatively slow [7].

Most researchers focus on methods such as the glass-phase connection, active metal brazing, and the MAX-phase connection. But, the MAX-phase joint requires high pressures during the brazing process, which is not suitable for engineering applications; the performance of the SiO_2_-Al_2_O_3_-Y_2_O_3_ glass-phase joint rapidly decreased after irradiation. Compared to the other methods, active metal brazing is one of the most convenient and widely used ceramic-joining methods due to its low cost, great flexibility, and high adaptability [8,9].

In recent years, many reports have revealed that an active element in the braze filler was essential to react with the inert ceramic surface and form a reaction layer, which was attributed to improving the wettability of the braze filler and the mechanical properties of the joints [10,11,12]. Different kinds of active fillers have been used for brazing SiC, including Ag-Cu-Ti alloy [13], nickel alloy [14], etc. Studies have shown that nickel alloy is an ideal kind of high-temperature filler [15,16]. The problem is that the reaction between Ni and SiC is usually violent and produces undesirable products such as graphite, which severely decreases the joints’ properties. Therefore, it is necessary to control the Ni/SiC reaction at the interface and reduce the formation of graphite [17,18]. Studies on the Ti active filler alloy such as Cu–10TiH_2_ indicates that the element Ti did not react with SiC at first and a proper buffer layer contributed to forming a good gradient transition of coefficient of thermal expansion, releasing residual stress and improving the shear strength of the joints [19]. Other studies have indicated that Ti can inhibit the reaction between Ni and SiC [20,21]. However, investigation of the transition mechanism is still lacking and the exact component point of the transition is also unknown.

In this paper, Ni-Ti brazing fillers with different compositions (NiTi + a Ni system) are used to join SiC ceramic. To find out the interfacial reaction transformation mechanism between Ni-Ti fillers and SiC, the thermodynamic analysis is introduced, and the filler’s composition during the transformation from the Ni/SiC reaction to the Ti/SiC reaction is calculated. The effect of the filler composition on the mechanical properties of SiC joints is also evaluated. The results outlined in this paper reveal the evolution of the microstructure, interface reactions, and properties of SiC joints brazed with Ni-Ti fillers, so as to reveal a possible joint technology for SiC_f_/SiC composite materials.

## 2. Research Methods

Pressureless sintered 6H-SiC ceramics, cut into small blocks with a dimension of 15 × 10 × 5 mm, were used as the bonding substrates. Before brazing, the faying faces of SiC were ground on a diamond grinding disc and then sonic cleaning was carried out for 30 min to remove the impurities. The final surface roughness was around Ra 0.8 μm. NiTi alloy powders (~50 μm) and different amounts of pure nickel powders (~1 μm, the purity was >99.9%) were evenly mixed with the 10 wt.% organic binders to form the filler paste. Three different compositions of NiTi + Ni mixed fillers were used, containing 15%, 30%, and 45% titanium, named Ni-15Ti, Ni-30Ti, and Ni-45Ti in this paper, respectively.

**Figure 1:**
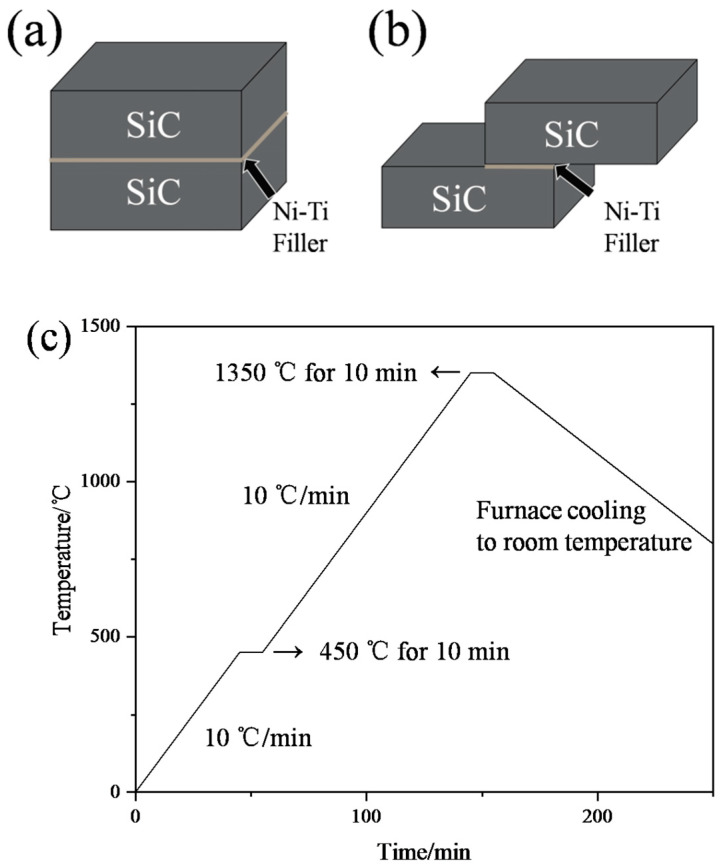
(**a**,**b**) Schematic diagrams of the SiC joints, and (**c**) curve of the brazing process.

The SiC sandwiched brazing couples were assembled in two ways, as shown in Figure 1a,b, and were used to observe the microstructure and test the mechanical properties. The couples were heated in a vacuum furnace (VQS-335, SZVAC, Shenyang, China), in which the vacuum pressure level was kept at ~7.0 × 10^−3^ Pa. As is shown in Figure 1c, the SiC brazed couples were first heated up to 450 °C at a rate of 10 °C/min and maintained for 10 min to obliterate the organic glue. Then, the couples were heated to 1350 °C at 10 °C/min and held for 10 min. The brazing temperature was chosen according to the melting point of NiTi alloy powder (1310 °C). Finally, the brazed joints were slowly cooled to room temperature in the furnace.

**Figure 2:**
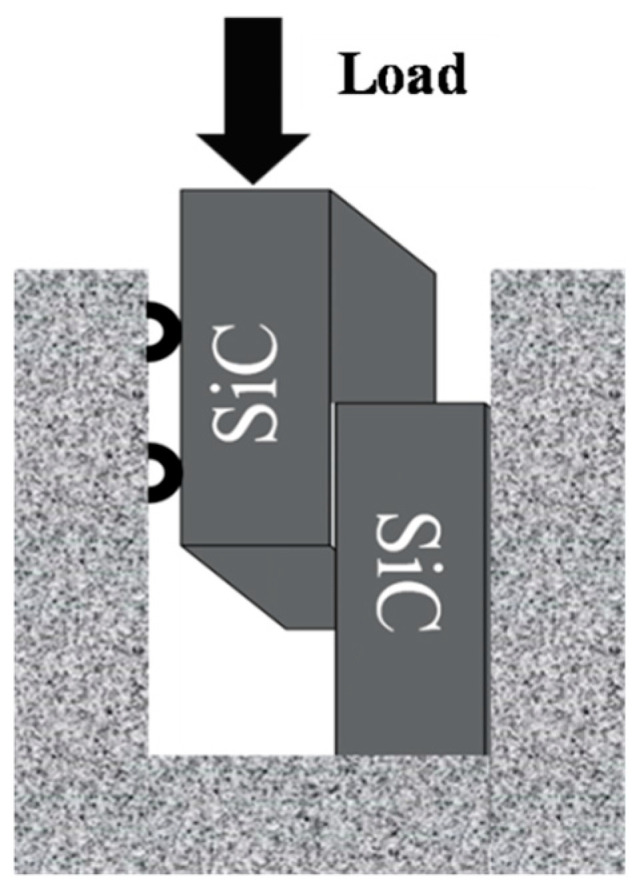
Schematic diagram of mechanical properties test.

The SiC joint microstructure of the brazing seam was observed by a scanning electron microscope (SEM), and the elemental composition was characterized by an energy dispersive spectrometer (EDS). The mechanical properties of the joints were calculated by their room-temperature shear strength, tested by an electronic universal test machine (RGX-M300, Reger Instrument, Shenzhen, China) at the rate of 0.5 mm/min. As is shown in Figure 2, the average shear strength of four SiC joints was tested at room temperature to characterize the mechanical properties of the SiC joints brazed by Ni-Ti fillers with different compositions. The fracture path and surface of each specimen was observed using a SEM (JSM-7500F, JEOL, Tokyo, Japan). The phases of the brazing seam were further confirmed by X-ray diffraction (XRD) from 20° to 80° by scanning the fracture surface.

## 3. Results and Discussion

### 3.1. Microstructure of the SiC/Ni-Ti/SiC Joints with Different Filler Compositions

**Figure 3:**
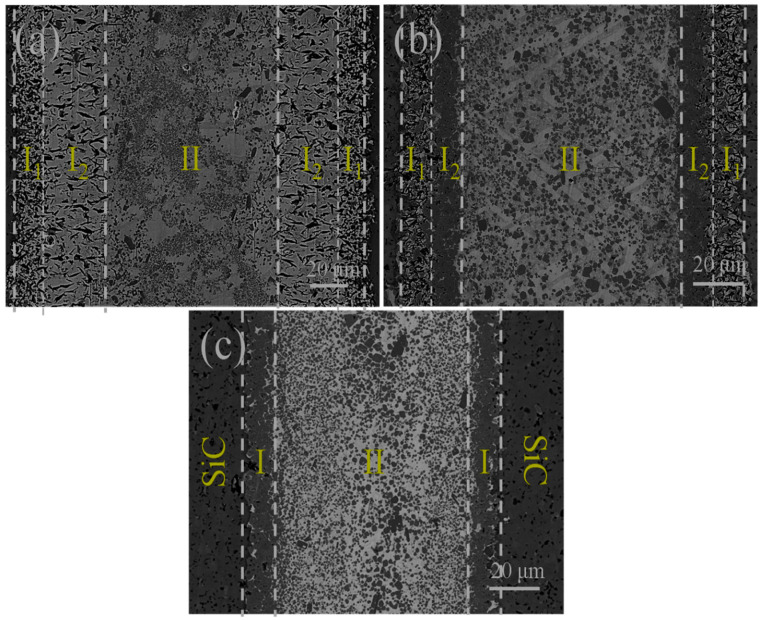
Microstructure of the SiC/Ni-Ti/SiC joints at 1350 °C for 10 min: (**a**) Ni-15Ti, (**b**) Ni-30Ti, and (**c**) Ni-45Ti.

Figure 3 shows the SEM images of the microstructure of SiC/Ni-Ti/SiC joints with different filler compositions at 1350 °C for 10 min. As is shown in Figure 3, all SiC ceramics were successfully joined, and cavities or thermal effects were not found in the brazing seam. All brazing seams can be divided into the interface layer (Zone I) and central area (Zone II).

**Figure 4:**
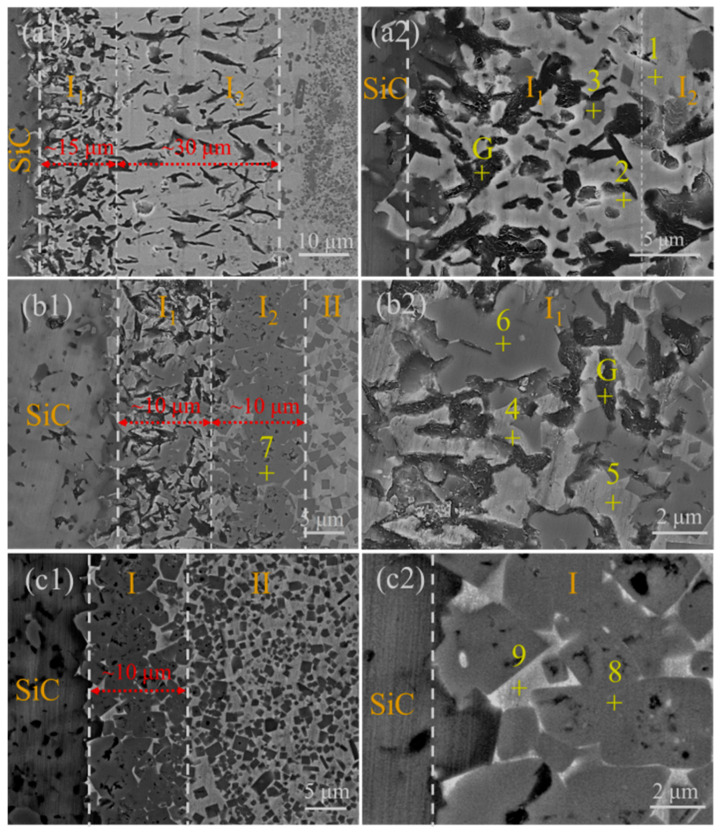
Microstructure of the interface area of the SiC/Ni-Ti/SiC joints at 1350 °C for 10 min: (**a1**) Ni-15Ti, (**a2**) positions of the points in Table 1, (**b1**) Ni-30Ti, (**b2**) positions of the points in Table 1, (**c1**) Ni-45Ti and (**c2**) positions of the points in Table 1.

**Figure 5:**
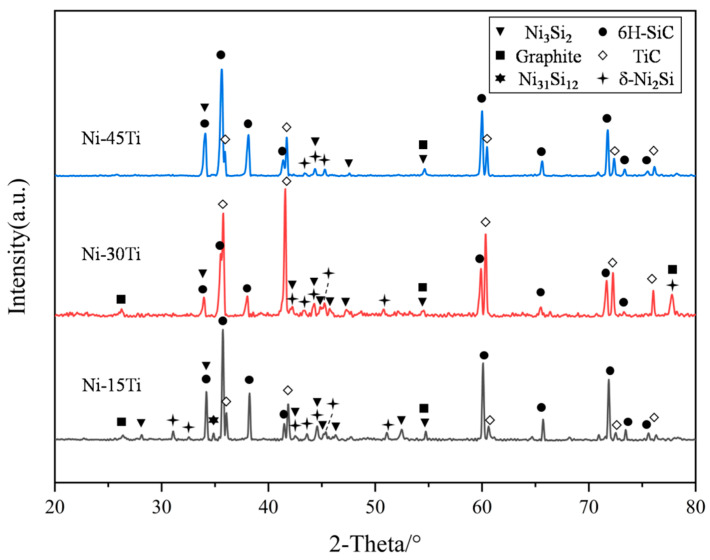
XRD patterns of the SiC joints brazed by different Ni-Ti fillers.

Figure 4 shows the microstructure of the interface layer. Combined with the XRD patterns of the interface shown in Figure 5 and the EDS results shown in Table 1, this indicates that the microstructure and width of the interface layer are obviously different with the change in the Ni-Ti fillers. To be specific, for SiC joints using the Ni-15Ti filler, the interface layer is the widest and composed of Ni_2_Si (Point 1), Ni_3_Si_2_ (Point 2), TiC particles (Point 3), and graphite (Point G). For SiC joints using the Ni-30Ti filler, the interface area consists of two layers. Zone I_1_ is a mixed reaction layer composed of three phases, including Ni_2_Si (Point 4), Ni_3_Si_2_ (Point 5), TiC (Point 6), and graphite (Point G). Zone I_2_ is a continuous TiC layer (Point 7). For SiC joints using the Ni-45Ti filler, graphite is no longer visually observed in the interface layer, and in the XRD pattern, graphite is only present at around 55 degrees with a very low intensity. So, the interface layer (Zone I) is only formed by the single TiC continuous layer (Point 8) with a small amount of residual Ni_2_Si (Point 9).

**Figure 6:**
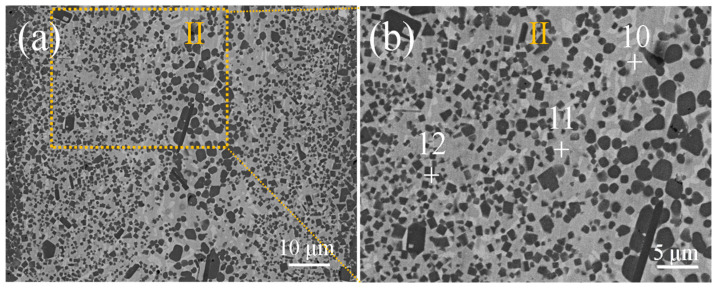
Microstructure of the central area of the SiC/Ni-45Ti/SiC joints at 1350 °C for 10 min. (**a**) SiC/Ni-45Ti/SiC joints; (**b**) positions of the points in Table 2.

For the central area (Zone II), the microstructure of the three joints is similar, and the typical phases are shown in Figure 6 and Table 2. The matrix structure is composed of Ni_2_Si (Point 10) and Ni_3_Si_2_ (Point 11), and a large number of TiC particles (Point 12) are distributed on the matrix.

### 3.2. Mechanical Strength of the SiC/Ni-Ti/SiC Joints

**Figure 7:**
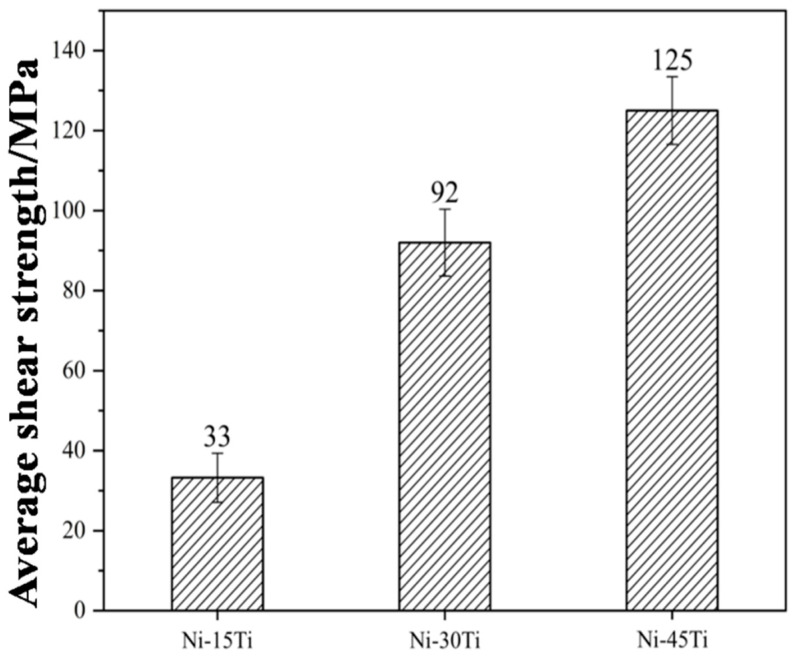
Average shear strength of SiC joints brazed by different Ni-Ti fillers.

As is shown in Figure 7, the shear strength of SiC joints using the different Ni-Ti fillers was determined at room temperature. The average shear strength of SiC joints using Ni-15Ti, Ni-30Ti, and Ni-45Ti fillers was 33 MPa, 92 MPa, and 125 MPa, respectively.

**Figure 8:**
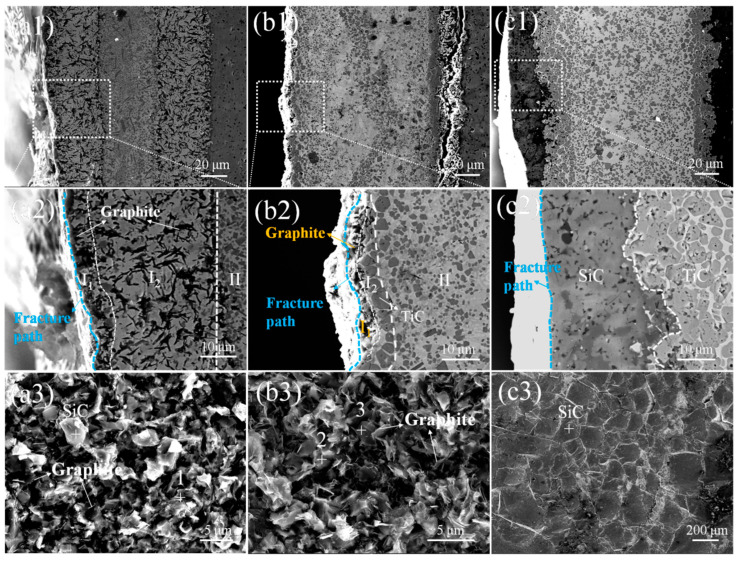
Microstructure of SiC joints after shear testing: (**a**) Ni-15Ti, (**b**) Ni-30Ti, and (**c**) Ni-45Ti. Here, (**a1**,**b1**,**c1**) and (**a2**,**b2**,**c2**) represent the morphology of the fracture path, and (**a3**,**b3**,**c3**) represent the microstructure of the fracture surface.

Figure 8 shows the images of the fracture path and fracture surface. For SiC/Ni-15Ti/SiC and SiC/Ni-30Ti/SiC joints, the crack propagates at the interface layer, and much graphite appears on the fracture surface, which suggests that the product of the interfacial reaction between Ni and SiC has a terrible effect on the joint strength. On the one hand, the graphite is equivalent to a large number of small hole defects. Once the external load is applied, the crack spreads rapidly, causing the joints to fracture at a low strength. On the other hand, the crack of the SiC/Ni-30Ti/SiC joints is hindered and deflected to other paths due to the presence of TiC in Zone I_1_, which acts as the strengthening phase, consumes more energy, and makes it obtain a higher strength than the SiC/Ni-15Ti/SiC joints.

The maximum average shear strength of 125 MPa was obtained when the Ni-45Ti filler was used. As is shown in Figure 8c, the fracture path of the SiC joints is located on the ceramics near the interface, indicating a high bonding strength between the brazing seam and the SiC ceramics [13]. A typical cleavage facet is observed on the fracture surfaces, and almost only SiC exists on the fracture surface. In addition to the stress relief of dispersed TiC particles in the central area [22,23], the high strength of the joint is mainly derived from the high joint interface between TiC and SiC, since the close interplanar spacing and good lattice matching between the TiC and SiC at the interface was proven by Yano et al. [24].

Shear tests of the SiC joints show a strong relationship between interfacial reaction products and joint strength. The transformation of the interface layer from the Ni-Si + graphite mixed layer to the TiC single layer significantly improves the joint strength. This suggests that there is an ideal composition of the Ni-Ti brazing filler, since samples with a composition of titanium larger than 45% cannot be obtained through this method.

### 3.3. Thermodynamic Analysis on the Interfacial Reaction Transformation Mechanism

From a thermodynamic point of view, a reaction occurs first if its Gibbs free energy is the lowest among all the reactions in the system. Based on the reaction equilibrium, by calculating and comparing the Gibbs free energy of the possible interfacial reaction in the brazing seam, the liquid phase composition of Ni-Ti alloy and SiC can be estimated when the transition from the Ni/SiC reaction to the Ti/SiC reaction occurs. According to the experimental results, there are two possibilities when 1 mol of Ni_1−*x*_Ti*_x_* liquid (labeled as *L*_0_, and *x* is the mole fraction of Ti) reacts with a minimal amount of *ε* mol SiC at 1350 °C. On the one hand, when the Ni/SiC reaction is dominant at the interface, C and the Ni-Ti-Si liquid with new compositions (labeled as *L*_1_) are formed. The reaction equation is expressed as

(1) $${\left({\mathrm{Ni}}_{1-x}{\mathrm{Ti}}_{x}\right)}_{0}+\varepsilon \langle \mathrm{SiC}\rangle \to \varepsilon \langle C\rangle +\left(1+\varepsilon \right){\left({\mathrm{Ni}}_{\left(1-x\right)/\left(1+\varepsilon \right)}{\mathrm{Ti}}_{x/\left(1+\varepsilon \right)}{\mathrm{Si}}_{\varepsilon /\left(1+\varepsilon \right)}\right)}_{1}$$

The Ni-Ti liquid is considered to be the ideal solution to simplify the analysis, ignoring the nucleation resistance, thermal effect, and volume effect. Therefore, the reaction Gibbs free energy of Equation (1) is written as

(2) $$\Delta G_{\mathrm{Ni}/\mathrm{SiC}}=\left(1+\varepsilon \right)G_{1}+\varepsilon G_{C}-\varepsilon G_{\mathrm{SiC}}-G_{0}$$

with

(3) $$\begin{array}{cc} G_{1} & =\frac{1-x}{1+\varepsilon }G_{\mathrm{Ni}}+\frac{x}{1+\varepsilon }G_{\mathrm{Ti}}+\frac{\varepsilon }{1+\varepsilon }G_{\mathrm{Si}}\\ & +RT\left[\frac{1-x}{1+\varepsilon }\ln\frac{1-x}{1+\varepsilon }+\frac{x}{1+\varepsilon }\ln\frac{x}{1+\varepsilon }+\frac{\varepsilon }{1+\varepsilon }\ln\frac{\varepsilon }{1+\varepsilon }\right] \end{array}$$

where *G*_0_, *G*_1_, *G*_SiC_, *G*_Si_, *G*_C_, *G*_Ni_, and *G*_Ti_ are the Gibbs free energy of *L*_0_, *L*_1_, SiC, Si, C, Ni, and Ti, respectively; the ideal gas constant R = 8.314 J·mol^−1^·K^−1^; and *T* is temperature.

On the other hand, when the Ti/SiC reaction is dominant at the interface, the active element, Ti, in the liquid phase reacts with SiC to form TiC and the Ni-Ti-Si liquid with the changed composition (labeled as *L*_2_). The reaction equation is expressed as

(4) $${\left({\mathrm{Ni}}_{1-x}{\mathrm{Ti}}_{x}\right)}_{0}+\varepsilon \langle \mathrm{SiC}\rangle \to \varepsilon \langle \mathrm{TiC}\rangle +{\left({\mathrm{Ni}}_{1-x}{\mathrm{Ti}}_{x-\varepsilon }{\mathrm{Si}}_{\varepsilon }\right)}_{2}$$

The reaction Gibbs free energy of Equation (4) is written as

(5) $$\Delta G_{\mathrm{Ti}/\mathrm{SiC}}=G_{2}+\varepsilon G_{\mathrm{TiC}}-\varepsilon G_{\mathrm{SiC}}-G_{0}$$

with

(6) $$\begin{array}{cc} G_{2} & =\left(1-x\right)G_{\mathrm{Ni}}+\left(x-\varepsilon \right)G_{\mathrm{Ti}}+\varepsilon G_{\mathrm{Si}}\\ & +RT\left[\left(1-x\right)\ln\left(1-x\right)+\left(x-\varepsilon \right)\ln\left(x-\varepsilon \right)+\varepsilon \ln\varepsilon \right] \end{array}$$

where *G*_2_ is the Gibbs free energy of *L*_2_, and *G*_TiC_ is the Gibbs free energy of TiC.

The Ni-Ti liquid and SiC are in thermodynamic equilibrium when Δ*G*_Ni/SiC_ = Δ*G*_Ti/SiC_. In other words, neither Equation (1) nor Equation (4) occurs between the liquid and SiC. Once the mole fraction of Ti or Ni changes, the thermodynamic equilibrium is broken. The liquid reacts with SiC according to either Equation (1) or Equation (4) to generate corresponding reaction products. Combining Equations (2) and (5), *f*(*x*) can be obtained as

(7) $$\begin{array}{cc} f\left(x\right) & =\Delta G_{\mathrm{Ni}/\mathrm{SiC}}-\Delta G_{\mathrm{Ti}/\mathrm{SiC}}\\ & =RT\left[x\ln x-\left(x-\varepsilon \right)\ln\left(x-\varepsilon \right)-\left(1+\varepsilon \right)\ln\left(1+\varepsilon \right)\right]\\ & +\varepsilon G_{\mathrm{Ti}}+\varepsilon G_{C}-\varepsilon G_{\mathrm{TiC}} \end{array}$$

**Figure 9:**
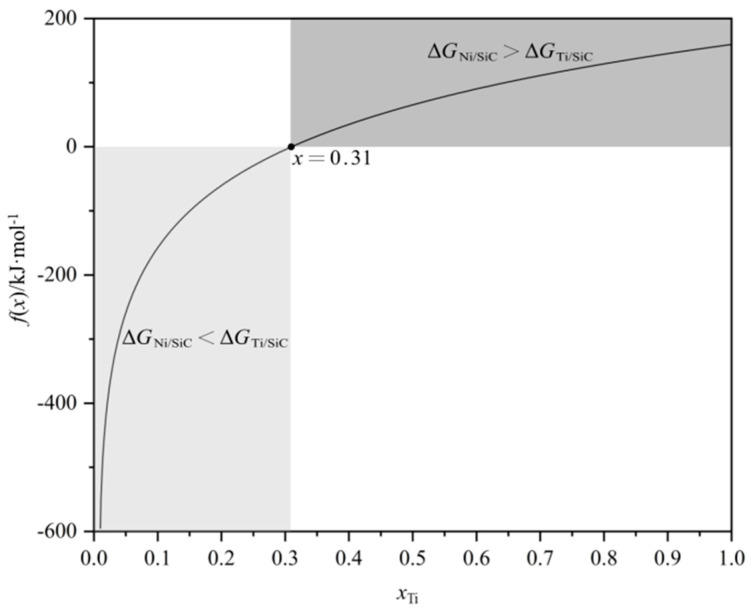
The diagram of *f*(*x*) = Δ*G*_Ni/SiC_ − Δ*G*_Ti/SiC_.

Finding the zero point and monotonicity of Equation (7) is crucial for analyzing the reaction’s transition. Therefore, *ε* = 0.01 is assumed, and *G*_Ti_ = −140,112 J/mol, *G*_C_ = −31,575.3 J/mol, and *G*_TiC_ = −140,112 J/mol are obtained at 1350 °C using the data provided by Du et al. [25]. The diagram of *f*(*x*) in the interval of (0, 1) is drawn as shown in Figure 9. The graph shows that *f*(*x*) monotonically increases in the interval of (0, 1), and *f*(*x*) = 0 at the point of 0.31. Based on the results, if *x*(Ti) is less than 0.31 (Δ*G*_Ni/SiC_ < Δ*G*_Ti/SiC_), only the Ni/SiC reaction will occur according to Equation (3), as is obtained in the experiment for Ni-15Ti. When the Ti content increases to near 0.31, neither the Ni/SiC nor the Ti/SiC reaction will happen in theory. However, the Ni_2_Si + graphite + TiC layer is generated when Ni-30Ti fillers are used in the actual experiment. It is speculated that the Ni/SiC reaction and Ti/SiC reaction may co-occur due to the fluctuation of Ni and Ti contents in the interface liquid phase. When *x*(Ti) is greater than 0.31 (Δ*G*_Ni/SiC_ > Δ*G*_Ti/SiC_), the Ti/SiC reaction dominates the interface, and the filler reacts with SiC to generate the single TiC layer according to Equation (6), as is obtained in the experiment for Ni-45Ti.

## 4. Conclusions

In this study, SiC ceramics were brazed by Ni-Ti fillers with different compositions at 1350 °C for 10 min to control the interfacial reaction. The effect of the fillers’ composition on the microstructure and mechanical properties is studied. Based on the thermodynamic calculation, the reaction transformation mechanism between Ni-Ti fillers and SiC ceramics is revealed, providing a theoretical reference for the composition design of Ni-based alloy fillers used for SiC ceramic brazing. The main conclusions of this paper are as follows:

(1) The Ti element can inhibit the Ni/SiC interface reaction in the process of brazing SiC ceramics with Ni-based fillers. The experimental results show that TiC replaces the Ni-Si compound and graphite as the only phase of the interface layer with an increase in Ti content.

(2) Graphite is harmful to the properties of the SiC joints. By eliminating graphite at the interface, the joints using a Ni-45Ti filler obtain the highest average shear strength of 125 MPa.

(3) The transformation of interfacial reaction products from Ni-Si + graphite + TiC to TiC is ascribed to the decrease in the Gibbs free energy of the Ti/SiC reaction. When the Ti content of the Ni-Ti fillers is higher than 31 at.%, Δ*G*_Ni/SiC_ > Δ*G*_Ti/SiC_, the Ti/SiC reaction preferentially occurs, and the TiC layer is generated at the interface. In other words, the Ni/SiC reaction is inhibited and the harmful graphite is eliminated.

## Figures and Tables

**Table 1:** EDS results of the points marked in Figure 4.

Filler	Point	Composition (at.%)				Possible Phases
		Ni	Ti	Si	C	
Ni-15Ti	1	37.0	0.3	18.6	44.1	Ni_2_Si
	2	34.0	0.1	21.9	43.9	Ni_3_Si_2_
	3	2.3	43.6	3.8	50.2	TiC
	G	0.9	0.5	1.5	97.0	Graphite
Ni-30Ti	4	52.9	1.3	25.3	20.5	Ni_2_Si
	5	50.1	-	29.9	20.0	Ni_3_Si_2_
	6	1.6	45.0	0.9	52.6	TiC
	G	0.9	0.5	1.5	97.0	Graphite
	7	-	44.8	1.4	53.8	TiC
Ni-45Ti	8	1.4	32.2	0.5	65.9	TiC
	9	41.4	4.0	22.1	32.4	Ni_2_Si

**Table 2:** EDS results of the points marked in Figure 6.

Filler	Point	Compositions (at.%)				Possible Phases
		Ni	Ti	Si	C	
Ni-45Ti	10	41.3	1.3	21.3	36.2	Ni_2_Si
	11	35.8	1.2	24.4	38.7	Ni_3_Si_2_
	12	1.7	31.0	1.2	66.1	TiC

## Data Availability

The original contributions presented in this study are included in the article. Further inquiries can be directed to the corresponding author.
